# Dynamical predictions of insular hubs for social cognition and their application to stroke

**DOI:** 10.3389/fnbeh.2014.00380

**Published:** 2014-11-04

**Authors:** Roberto Limongi, Ailin Tomio, Agustin Ibanez

**Affiliations:** ^1^UDP-INECO Foundation Core on Neuroscience (UIFCoN), Diego Portales UniversitySantiago, Chile; ^2^Laboratory of Experimental Psychology and Neuroscience (LPEN), Institute of Cognitive Neurology (INECO), Favaloro UniversityBuenos Aires, Argentina; ^3^Universidad Autónoma del CaribeBarranquilla, Colombia; ^4^National Scientific and Technical Research Council (CONICET)Buenos Aires, Argentina; ^5^Centre of Excellence in Cognition and its Disorders, Australian Research Council (ACR)Sydney, Australia

**Keywords:** insula, emotion, social cognition, stroke, effective connectivity, predictive coding

## Abstract

The insular cortex (IC) is considered a rich hub for context-sensitive emotions/social cognition. Patients with focal IC stroke provide unique opportunities to study socio-emotional processes. Nevertheless, Couto et al. (2013b) have recently reported controversial results regarding IC involvement in emotion and social cognition. Similarly, patients with similar lesions show high functional variability, ranging from almost totally preserved to strongly impaired behavior. Critical evidence suggests that the variability of these patients in the above domains can be explained by enhanced neuroplasticity, compensatory processes, and functional remapping after stroke. Therefore, socio-emotional processes would depend on long-distance connections between the IC and frontotemporal regions. We propose that predictive coding and effective connectivity represent a novel approach to explore functional connectivity and assess compensatory, contralateral, and subsidiary network differences among focal stroke patients. This approach would help explain why socio-emotional performance is so variable within this population.

## The insula: a rich hub for social cognition

The insular cortex (IC), a brain region localized deep in the lateral sulcus, has been recently considered an interoceptive region (Penfield and Faulk, 1955; Greenspan et al., 1999; Aziz et al., 2000; Harper et al., 2000; Cameron and Minoshima, 2002; Craig, 2002; Critchley et al., 2004). The IC is also regarded as a fundamental substrate of emotional experience and social cognition (Couto et al., 2013b). Several studies have examined the role of the IC in the recognition, experience, and imagination of basic emotions (Jabbi et al., 2008; Sprengelmeyer et al., 2010), as well as in empathy and social cognition (Bernhardt and Singer, 2012; Decety et al., 2012a; Koban and Pourtois, 2014). Connectivity measures have shown anterior IC (aIC) involvement in different networks subserving cognitive, homeostatic, and socio-emotional processes (Kelly et al., 2012). For its own part, the right aIC plays an integrative role in coordinating awareness of body feelings (Craig, 2002), contextual social clues (Amoruso et al., 2011; Bernhardt and Singer, 2012; Ibañez and Manes, 2012b), and emotional salient stimuli (Seeley et al., 2007; Ibañez et al., 2010). The IC role in coordinating emotions and social cognition depends on a wide arrangement of structural connections. Briefly, regions ubiquitously activated in social cognition tasks, such as the medial prefrontal cortex (mPFC; including the anterior cingulate cortex, ACC) and the temporo-parietal junction (TPJ), are connected with the IC. These regions connect distant modules within the network and offer links to other functional networks (Liang et al., 2013; van den Heuvel and Sporns, 2013). Therefore, IC has been acknowledged as part of a “rich hub” that integrates global information within the brain (Kelly et al., 2012).

Internal body signaling has been proposed to trigger modulations of emotional processing and social cognition (Melloni et al., 2013; Couto et al., 2014; Sedeño et al., 2014; Uddin et al., 2014). The right aIC has been specifically assumed to redirect attention in response to emotionally salient stimuli (salience network/ventral attention system; Fox et al., 2006; Seeley et al., 2007; Corbetta et al., 2008; Eckert et al., 2009; Menon and Uddin, 2010; Nelson et al., 2010; Cirneci, 2011). The aIC has also been implicated in social emotions and social cognition (Singer et al., 2004; Keysers and Gazzola, 2007; Saarela et al., 2007; Caruana et al., 2011; Decety et al., 2012b; Ibañez and Manes, 2012b; Melloni et al., 2014). Patients with frontotemporal dementia have shown deficits in empathy (Baez et al., 2014b; see also similar results in early neurodegeneration: Baez et al., 2013a), moral cognition (Baez et al., 2014a) and mentalizing processes associated with IC degeneration (Couto et al., 2013a). In addition, meta-analytic evidence has revealed IC involvement in non-story-based ToM studies (Mar, 2011). More straightforwardly, several models propose that the contextual association of environmental and internally driven emotional signals trigger specific social responses (Decety and Jackson, 2004; Corbetta et al., 2008; Adolphs, 2009; Lamm et al., 2010; Lamm and Singer, 2010; Ibañez and Manes, 2012a; Baez and Ibanez, 2014; Sedeño et al., 2014). The IC is considered a relay region for the integration of bottom-up interoceptive/emotional signals and higher-level regulation of information flow (Ibañez and Manes, 2012a; Gu et al., 2013). In addition, predictive coding offers a new view of emotion as interoceptive inference (Seth et al., 2012). In brief, the aIC is a suitable hub to integrate internal body signals, emotion, and social cognition processes: it conveys a sense of the organism’s homeostatic status (Craig, 2003) and is ubiquitously engaged in affective-cognitive networks (Kurth et al., 2010; Uddin et al., 2014). As previously suggested (Uddin et al., 2014), the aIC can be considered a critical site for the integration of internal information with emotional and social stimuli.

## Controversies about insular specificity for emotion and social cognition: lesion studies

Despite the evidence summarized above, data regarding specific IC involvement in socio-emotional processes are controversial. Results have been particularly inconsistent in stroke-patient studies. Patients with focal stroke provide a unique framework to study brain function, as they afford insights into how crucial an area is fora specific function (Rorden and Karnath, 2004).

Disgust has been related to IC. In this sense, Calder et al. (2000) and Adolph et al. (Calder et al., 2000; Adolphs et al., 2003) reported patients with IC damage who showed deficits in both the recognition and experience of disgust. Such findings support the role of the insula in the perception of aversive emotions. Moreover, the right insula is associated with negative emotions in general, and disgust in particular (Shapira et al., 2003; Sambataro et al., 2006; Henley et al., 2008; Chen et al., 2009; Ruiz et al., 2013). Indeed, recognition of disgust requires the integrity of right-sided structures (Adolphs et al., 2000). With the exception of some prefrontal areas (Harmon-Jones, 2004), most studies show a left-side participation in positive emotion impairments, and right-side involvement in negative emotion deficits (for reviews, see Adolphs, 2002; Brown et al., 2011). However, Straube et al. (2010) reported spared disgust recognition and emotion experience in a patient with a right IC stroke.

Although disgust recognition studies differ in the side of the lesion (left in Calder’s study and right in Straube’s study), the right IC (whose lesion did not impair disgust recognition in Straube’s) is supposed to index negative emotions and disgust, as reported in several other studies (Adolphs et al., 2000; Shapira et al., 2003; Sambataro et al., 2006; Henley et al., 2008; Chen et al., 2009; Ruiz et al., 2013; for reviews, see Adolphs, 2002; Brown et al., 2011). Thus, stroke evidence regarding disgust recognition remains controversial; these results call for an explanation of the impairments’ variability.

Similarly, the IC has been identified as part of a set of regions implicated in the processing of empathy (Leigh et al., 2013). Although functional imaging studies consistently show aIC activation in affective empathy and moral tasks (Bzdok et al., 2012), temporal lobe lesions (and not only IC lesions) are frequently associated with impairments in affective empathy. For example, 90% of the patients studied by Leigh et al. (2013) (all with damage to the IC and the temporal lobe) presented deficits in affective empathy tasks; and the only patient with spared affective empathy had damage in the IC but not in the temporal lobe. One alternative explanation is that this patient’s lesion was too small to induce an effect. Despite this possibility, the error rate during task performance was not directly associated with injured IC volume. For example, patients 1 and 7 showed similar error rates although they featured 77% and 23.6% of insular tissue damage, respectively presented similar performance; in turn, both patients 9 and 8 presented a 100% error rate, although their insular lesion involvement was 96% and 0%, respectively. Notably, all patients with insular lesions that presented impaired empathy also exhibited temporal lesions; in fact, damage to temporal regions (anterior temporal pole, superior temporal gyrus, middle temporal gyrus) was the best predictor of performance. Therefore, it appears that IC damage itself does not explain affective empathy impairments.

Regarding the lateralization of IC in the empathy studies, several reports have shown a general right predominance (Bodini et al., 2004; Brüne and Brüne-Cohrs, 2006; Shamay-Tsoory, 2011; Leigh et al., 2013). Both right anterior insula and right temporal pole atrophy have been associated with impaired affective empathy in neurodegenerative disease (Rankin et al., 2005; Kipps and Hodges, 2006; Narvid et al., 2009; Lee et al., 2012). However, a meta-analysis of previous studies has not found a specific lateralization of empathy within the insula (Lamm et al., 2011). In addition, another activation likelihood meta-analysis of 112 experiments on affective empathy reported a significant activation in the bilateral anterior insula in association with empathy tasks across studies (Bzdok et al., 2012). Thus, a bilateral participation of the insula in empathy is more expected.

Note, however, that the anatomical position and vascular supply (i.e., from the middle cerebral artery, MCA) of the IC renders focal damage extremely infrequent in clinical practice (Ibañez et al., 2010). For example, Cereda et al. (2002) reported that out of 4,800 stroke patients only four had a truly isolated IC infarction. Therefore, it remains unclear whether the socio-emotional changes observed in stroke patients with damage to the MCA territory actually stem from focal IC lesions. The controversial results obtained in lesion studies might reflect both the relative involvement of focal IC lesions and the severity of the damage sustained by insular connections (Ibañez et al., 2010). Incidentally, similar controversies surround other brain structures. For instance, frontal lobe patients with similar lesions also show high cognitive variability, ranging from almost totally preserved to strongly impaired performance in multiple domains, including both emotion recognition and social cognition (Mesulam, 1986; Ibañez and Manes, 2012a).

## Disentagling the insula from its connections in focal stroke studies

In a pioneering double case report, Couto et al. (2013b) distinguished the role of the IC from that of its connections in social emotions, higher order social cognition, and emotion recognition (especially, negative emotions and, in particular, disgust). The peculiarity of the lesions allowed the authors to go beyond previous studies. Whereas one subject had a very rare focal IC lesion (without any subcortical impairment), the other had a subcortical (striatal) focal stroke affecting the connections between the IC and frontotemporal regions. Both patients and a sample of matched controls were administered neuropsychological and affective screening questionnaires, an emotional inference disambiguation task (using social contextual clues, see also Baez and Ibanez, 2014), a battery of multimodal basic emotion recognition tests, an empathy task, and a theory-of-mind task. The insular lesion patient showed spared emotion recognition and social emotions (although response times were delayed in a subset of tasks). Surprisingly, this patient outperformed the controls in the prosody test and in recognition of disgust (the emotion believed to critically depend on the IC). In these tasks, the patient responded faster and more accurately than controls. Conversely, the subcortical lesion patient showed strong impairments in multimodal aversive emotion recognition (including disgust). The patient also displayed delayed response times and deficits in empathy and context-driven emotion inference.

These unexpected results suggest that IC-related networks, as opposed to the IC itself, are associated with negative emotional processing and social cognition. Thereby, the IC would play a critical role in the processing of emotion and social cognition, but only within the context of its frontotemporal connections. Thus, preserved emotion recognition and social cognition in insular patients, as well as the report of transitory post-stroke deficits in empathy and other social cognition domains (Eslinger et al., 2002), may reflect enhanced neuroplasticity and successful functional remapping of the IC-frontotemporal network after IC stroke (Couto et al., 2013b).

Alternatively, the deficits of the subcortical patient could reflect weakened interoceptive activity in the dorsal posterior insula. Although deficits in interoceptive awareness may partially explain the patient’s performance, there is evidence to the contrary. In a subsequent study (Couto et al., under review), we assessed interoceptive sensitivity in the same two patients and found that interoceptive-related behavior was differentially impaired in each case: the insular lesion affected cardiac interoceptive measures whereas the subcortical lesion yielded preserved performance. Thus, the subcortical patient’s impairments in social cognition and negative emotion cannot be explained by canonical interoceptive signals running through autonomous (vagal) pathways. These factors notwithstanding, further studies would elucidate the relationship between interoceptive sensitivity and social cognition in subcortical lesions.

The neuroanatomical and behavioral evidence offered by Couto et al. (2013b) also suggest that frontotemporal networks must be spared for intact emotional processing and social cognition (Ibañez and Manes, 2012a; Couto et al., 2013a; Melloni et al., 2014). Affective and cognitive information may be sent to the IC via subcortical tracts affording links to other nodes within the social-emotional network. Therefore, we suggest that social processes (from inference of emotions to empathy and moral cognition) depend on IC connections with frontal and temporal subcortical structures (Baez et al., 2012, 2013b, 2014a,b; Ibañez and Manes, 2012b; Couto et al., 2013a; Ibáñez et al., 2013; Lavin et al., 2013; Escobar et al., 2014; Melloni et al., 2014). In this view, the behavioral variability found in clinical patients may reflect diverse lesions affecting varied loci and their specific directional relations along this vast network.

## A predictive coding framework for insular hubs

The predictive coding approach is gaining acceptance within the neuroscience community (Friston, 2012). Predictive coding theory rests upon well-defined principles: The brain is an inference machine which creates top-down predictions on the causes of sensory inputs and updates such predictions via bottom-up prediction errors (PEs, the differences between actual and expected outcomes). Top-down predictions work via feedback information that minimizes PEs (Friston and Kiebel, 2009; Friston et al., 2010). Hierarchical minimization of PEs constitutes the core of predictive coding. PEs not minimized at a lower level in the hierarchy (e.g., primary visual cortex) pass to the next level in the hierarchy (e.g., associative areas). The process continues until PEs are completely minimized (Kiebel et al., 2008; Friston and Kiebel, 2009).

Predictive coding principles apply not only to basic brain function but also to social cognition (Brown and Brüne, 2012). Social information would serve modulatory purposes between bottom-up and top-down information (Brown and Brüne, 2012). More specifically, the IC has been conceived as a comparator locus in which interoceptive-related PE could be minimized (Seth and Critchley, 2013). Thus, interoceptive feeling states (emotions) can emerge from inferred predictive models of interoceptive afferents (Seth, 2013). Although this theoretical proposal seems attractive, it implies a methodological challenge for a suitable predictive-coding based research program: designing an experimental paradigm capable of generating PE that are not minimized at lower sensory regions. Put simply, based on the tenets of a hierarchical minimization process, PE that would be minimized in the IC must not have been explained away in lower cortical regions. We are currently working towards the development of such an experimental paradigm. In sum, predictive coding appears to be a suitable theoretical and methodological framework to study effective connectivity between the IC and related regions (Critchley and Seth, 2012; Seth et al., 2012).

Within the predictive coding approach, we could conceptualize the fronto-temporo-insular network as a hierarchy of connections. This hierarchy of connections accommodates a more parsimonious explanation and further hypotheses on previous bewildering findings. Specifically, the hypothesis that striatal-insular projections would convey social and affective information is consistent with the role of PEs and the hierarchical PE minimization process. PEs associated with negative emotions, empathy, and affective arousal, are conveyed to the IC and more frontal regions (higher in the hierarchy) by subcortical areas (lower in the hierarchy). PE-signals affecting the insular region would modulate connections, whereas PE-signals targeting more frontal areas would reach minimization.

The above hypothesis suggests a two-sided explanation of the behavioral effects reported by Couto et al. On the one hand, PEs and response times are strongly correlated (Feldman and Friston, 2010), which would account for the longer response times yielded by the insular patient when recognizing emotions. PE-related signals do not directly affect insular neurons; they modulate connections converging in the insula (Limongi et al., 2013). Therefore, damaged IC would not affect negative emotion recognition on the assumption of intact extrinsic connections. On the other hand, striatal/temporal lesions would limit the PE-signals modulating connections which converge in the IC. Moreover, PEs originating in the striatum would not reach higher regions in the hierarchy. Thus, damage to the striatum and other temporal sites would limit the PE minimization process. Behaviorally, the performance in negative emotion recognition would decrease in striatal patients.

**Figure 1:**
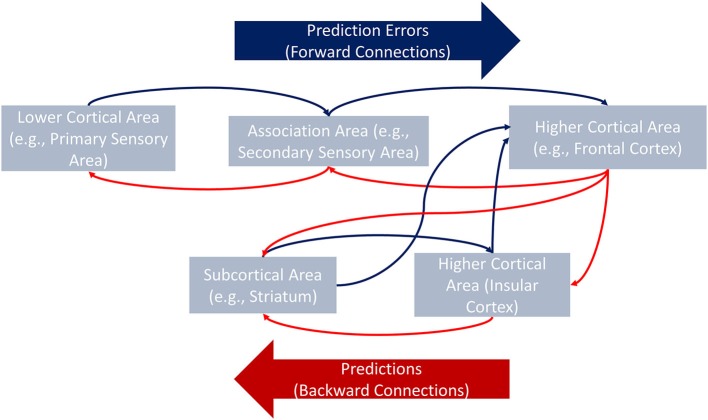
**General framework of the hierarchical minimization process of PEs**. Via backward connections, higher cortical areas convey hypotheses about, for example, the causes of the interoceptive information to lower cortical or subcortical areas (e.g., from the frontal cortex to the IC). Each level of the hierarchy conveys a specific prediction to the closest lower area in which canonical intrinsic interactions minimize the difference between the expected and the actual information (i.e., PE). When PEs are not minimized at a given level (e.g., in the striatum), they are passed along to the next level (e.g., the IC) which receives new predictions from the top of the hierarchy (the frontal cortex). The process continues upstream until the PEs are completely minimized.

This model (Figure 1) gives rise to distinct predictions regarding differences between insular patients and controls. In the former population, signals associated with longer response times would more strongly modulate connections converging in the IC (e.g., basal ganglia, ACC, orbitofrontal cortex, inferior frontal gyrus, and dorsolateral prefrontal cortex). The preserved contralateral side of the insula would still receive this modulatory information, but it would process such information less efficiently. Note that the explanations and hypotheses presented in this section refer to modulatory activity in between-region connections. Testing these hypotheses, therefore, requires methods beyond conventional functional connectivity (coactivity) analyses typically reported in the neuroscientific literature. One such method is effective connectivity analysis.

## Effective connectivity as a new approach to characterize lesion patient variability

Whereas functional connectivity describes coactivity between two or more brain regions, effective connectivity describes how the regions connect and how other regions modulate the connections. Dynamic causal models (DCM; Friston, 2007) comprise a biologically reliable strategy to specify driving inputs, modulatory inputs, and between-region connections that give rise to neurophysiological data (e.g., hemodynamic and electrophysiological responses). An innovative translational approach consists in pairing experimental conditions and group-specific covariates with the connections as well as driving and modulatory inputs. By means of DCM, the researcher can then specify competing hypotheses on the effects of the conditions/covariates. Optimal parameters representing the effects can be estimated through bilinear differential equations. Finally, using Bayesian model selection strategies (Stephan et al., 2009), the researcher can select the model or family of models (Penny et al., 2010; Rosa et al., 2012) that best account for the physiological data.

Social and emotional contexts probably make evident the largest variability among individuals. Effective connectivity offers a fine level of sensitivity to account for such variability. Although other effective connectivity approaches (e.g., independent multiple-sample greedy equivalence search (IMaGES; Ramsey et al., 2010) have been used in the field of social cognition (Hanson et al., 2013), DCM are more biologically reliable to identify interregional connectivity and describe how signals (such as PE) modulate the connections. If we can estimate which parameters describe an experimental effect in a control group, we can also describe the within- and between group variability in our parameters of interest. Moreover, by comparing parameters values, we could describe longitudinal effects of clinical, neuropsychological, and pharmacological interventions. We could also describe how the parameters vary during the evolution of neurodegenerative diseases affecting similar fronto-temporo-insular connections (e.g., frontotemporal dementia). These strategies have recently yielded promising results in research on epilepsy (Daunizeau et al., 2012) and Parkinson’s disease (Kahan et al., 2012).

## Conclusions

Current descriptions of socio-emotional impairments triggered by insular stroke (and related frontotemporal lesions) suggest high variability in the patients’ performance. This variability can be interpreted in terms of preserved/affected hubs connecting the lesion sites with other ipsilateral regions and with their contralateral regions. Here, we have proposed that effective connectivity analysis constitutes a novel approach to examine functional connectivity and explain why only some stroke patients are impaired in these domains. Specifically, effective connectivity studies allow us to assess compensatory, contralateral, and subsidiary networks. Moreover, given the existence of transitory post-stroke deficits in empathy and other social cognition domains, this approach may be used to test the functional-remapping hypothesis in cases of IC damage and similar hypotheses in patients with lesions to other brain regions. Dynamic causal models of effective connectivity may provide an opportunity to achieve “effective” cross-talk between clinicians and basic neuroscientists (Kahan and Foltynie, 2013). By means of effective connectivity studies, we may be able to understand why patients with similar lesions perform differently (i.e., very well or very badly) in emotional, social, and cognitive domains.

## Conflict of interest statement

The authors declare that the research was conducted in the absence of any commercial or financial relationships that could be construed as a potential conflict of interest.
